# Prevalence and factors associated with suicidal ideation and suicidal behavior among police personnel in Kerala, India: a cross-sectional study

**DOI:** 10.3389/fpubh.2026.1949658

**Published:** 2026-10-16

**Authors:** Adithya Sasi, Rashita Ravi, Jeby Jose Olickal, Kavumpurathu Raman Thankappan

**Affiliations:** Department of Public Health, Amrita School of Medicine, Amrita Vishwa Vidyapeetham, Kochi, Kerala, India

**Keywords:** behavior, cross-sectional study, C-SSRS, Kerala, occupational stress, police personnel, social support, suicidal ideation

## Abstract

**Introduction:**

Although police personnel are exposed to substantial occupational stress and Kerala has one of the highest suicide rates in India, evidence on suicidal ideation and suicidal behavior among police personnel in the state remains limited. This study estimated the prevalence of suicidal ideation and suicidal behavior and identified their associated factors among police personnel in Kerala, India.

**Methods:**

A cross-sectional study was conducted among 400 police personnel (mean age 37.94 years; 81% men), from Civil Police Officers (CPOs) to Inspectors, recruited from 15 police stations in Ernakulam and Malappuram districts of Kerala using a multistage cluster sampling approach. Data were collected using a self-administered structured questionnaire. Suicidal ideation and suicidal behavior during the past 12 months were assessed using the Columbia-Suicide Severity Rating Scale (C-SSRS), with the reference period adapted to the past 12 months. Survey-weighted generalised linear models with a Poisson distribution and log link were used to estimate adjusted prevalence ratios (aPRs) and 95% confidence intervals (CIs) for factors associated with suicidal ideation and suicidal behavior.

**Results:**

The survey-weighted prevalence of suicidal ideation was 9.39% (*n* = 36; 95% CI: 6.18–14.01%), while 4.11% (*n* = 14; 95% CI: 1.68–9.70%) reported suicidal behavior during the past 12 months. Actual suicide attempts were reported by 1.47% (*n* = 6; 95% CI: 0.66–3.25%). Among participants with suicidal ideation, 13.56% (*n* = 6) reported active suicidal ideation with a specific plan and intent. A higher prevalence of suicidal ideation was observed among participants aged >36 years (aPR: 2.90; 95% CI: 1.31–6.38), CPOs (aPR: 2.31; 95% CI: 1.59–3.37), those working >12 h per day (aPR: 1.67; 95% CI: 1.05–2.65), those with a previous psychiatric illness (aPR: 1.67; 95% CI: 1.19–2.35), and those reporting workplace violence or harassment (aPR: 3.16; 95% CI: 1.11–9.04). A higher prevalence of suicidal behavior was observed among participants with >15 years of service (aPR: 13.82; 95% CI: 5.00–38.19), CPOs (aPR: 12.32; 95% CI: 6.12–24.80), those with a previous psychiatric illness (aPR: 5.94; 95% CI: 2.58–13.66), and those reporting workplace violence or harassment (aPR: 13.81; 95% CI: 2.71–70.34).

**Conclusion:**

Nearly one in ten police personnel reported suicidal ideation and approximately one in twenty-five reported suicidal behavior during the past 12 months. The findings highlight the need for workplace mental health strategies addressing occupational stressors and improving access to confidential mental health support among police personnel.

## Introduction

Suicide remains a major global public health problem, with more than 720,000 deaths occurring annually worldwide, nearly three-fourths of which occur in low- and middle-income countries (1). Suicidal behavior includes suicidal ideation, suicide planning, suicide attempts, and completed suicide, all of which are associated with substantial morbidity and increased risk of future suicide mortality (2–4). Early identification of individuals experiencing suicidal thoughts or behaviors is therefore central to suicide prevention efforts (1, 4).

India contributes significantly to the global burden of suicide, with considerable regional variation in suicide rates influenced by sociocultural and economic factors (5, 6). The Indian state of Kerala presents a unique context, as the state continues to report relatively high suicide rates despite favorable health and literacy indicators (6, 7).

Police personnel are particularly vulnerable to mental health problems because of frequent exposure to traumatic events, occupational stress, long working hours, shift duties, violence, and organizational pressures (8–10). Mental health stigma and concerns regarding professional consequences may further discourage help-seeking among police personnel (11). Previous studies and reviews have identified occupational stress, depression, burnout, trauma exposure, substance misuse, and poor social support as important factors associated with suicidal ideation and suicidal attempts among police personnel (12–15).

In India, policing is primarily organized at the state level, with each state maintaining its own police force under the administrative control of the state government. The police force follows a hierarchical rank structure comprising constabulary, subordinate officer, and senior officer ranks. In Kerala, the police force comprises approximately 57,000 personnel and is organized into police districts, city police Commissionerate’s, specialized units, and individual police stations. At the police-station level, the hierarchy relevant to the present study includes Civil Police Officer (CPO), Senior Civil Police Officer (SCPO), Assistant Sub-Inspector (ASI), Sub-Inspector (SI), and Inspector of Police.

In India, research among police personnel has largely focused on occupational stress, burnout, anxiety, depression, and coping mechanisms (16–18). Evidence from Kerala has demonstrated a significant association between operational stress and suicidal ideation among police personnel (17). However, evidence on the prevalence and associated factors of suicidal ideation and suicidal behavior among police personnel in Kerala remains limited. Kerala provides an important context for such investigation because of its high population-level burden of suicide and the distinctive occupational and organizational environment in which police personnel work, including long working hours, frequent public interaction, and high societal expectations (6, 18). Context-specific evidence is therefore needed to better characterize suicidal ideation and suicidal behavior among police personnel and to inform locally relevant preventive strategies. Therefore, this study aimed to estimate the prevalence of suicidal ideation and suicidal behavior during the past 12 months and identify associated sociodemographic, occupational, health-related, and psychosocial factors among police personnel from selected police stations in Kerala.

## Materials and methods

### Study design

A cross-sectional study was conducted among police personnel in Kerala, India.

### Setting and population

The study was conducted in two districts of Kerala: Malappuram and Ernakulam. The study population comprised police personnel ranging from Civil Police Officer (CPO) to Inspector of Police serving in the selected police stations. Eligible participants were police personnel who had completed at least 6 months of continuous service in their current posting and provided written informed consent. Personnel who self-reported a psychiatric illness of sufficient severity to interfere with their ability to understand or independently complete the questionnaire were excluded. This eligibility criterion was assessed based on participant self-report; no participant was excluded on this basis.

### Study duration

This study was conducted between September 2025 and May 2026. Data collection was carried out between December 2025 and February 2026.

### Sample size

The sample size was calculated using OpenEpi Version 3.0, assuming a prevalence of 12-month suicidal ideation of 12.5% (19), a 95% confidence level, an absolute precision of 5% and a design effect of 2 to account for the cluster sampling design. The calculated minimum required sample size was 337. After adjusting for an anticipated non-response rate of 15%, the final sample size was increased to 400 participants.

### Sampling

A multistage cluster sampling approach was used. In the first stage, two police administrative settings in Kerala were selected to represent different geographical regions of the state: Malappuram District Police (northern Kerala) and Kochi City Police Commissionerate, Ernakulam (central Kerala). These administrative settings were treated as strata in the survey analysis. In the second stage, police stations were selected by simple random sampling within each stratum. Eight of the 31 police stations under Malappuram District Police (Nilambur, Edakkara, Wandoor, Kottakkal, Tirurangadi, Vengara, Pandikkad, and Perinthalmanna) and seven of the 27 police stations under Kochi City Police Commissionerate (Cheranalloor, Eloor, Ernakulam Central, Ernakulam Town North, Vanitha, Fort Kochi, and Elamakkara) were selected. These 15 police stations constituted the primary sampling units (PSUs), with station-level selection probabilities of 8/31 and 7/27 in Malappuram and Kochi, respectively.

The selected police stations had a combined personnel strength of 792, comprising 418 personnel in Malappuram and 374 in Kochi. In the final stage, eligible personnel who were available during the data-collection visits were invited to participate until the predetermined sample allocation for each administrative setting was achieved. As recruitment at the individual level was based on availability rather than random selection, exact individual-level selection probabilities could not be determined. To account for unequal sampling fractions across the selected police stations, station-specific sampling weights were calculated based on the personnel strength and number of participants recruited from each station and incorporated into the survey analysis. A total of 400 participants were enrolled, with 200 participants from each administrative setting. The detailed sampling process is presented in Figure 1.

**Figure 1:**
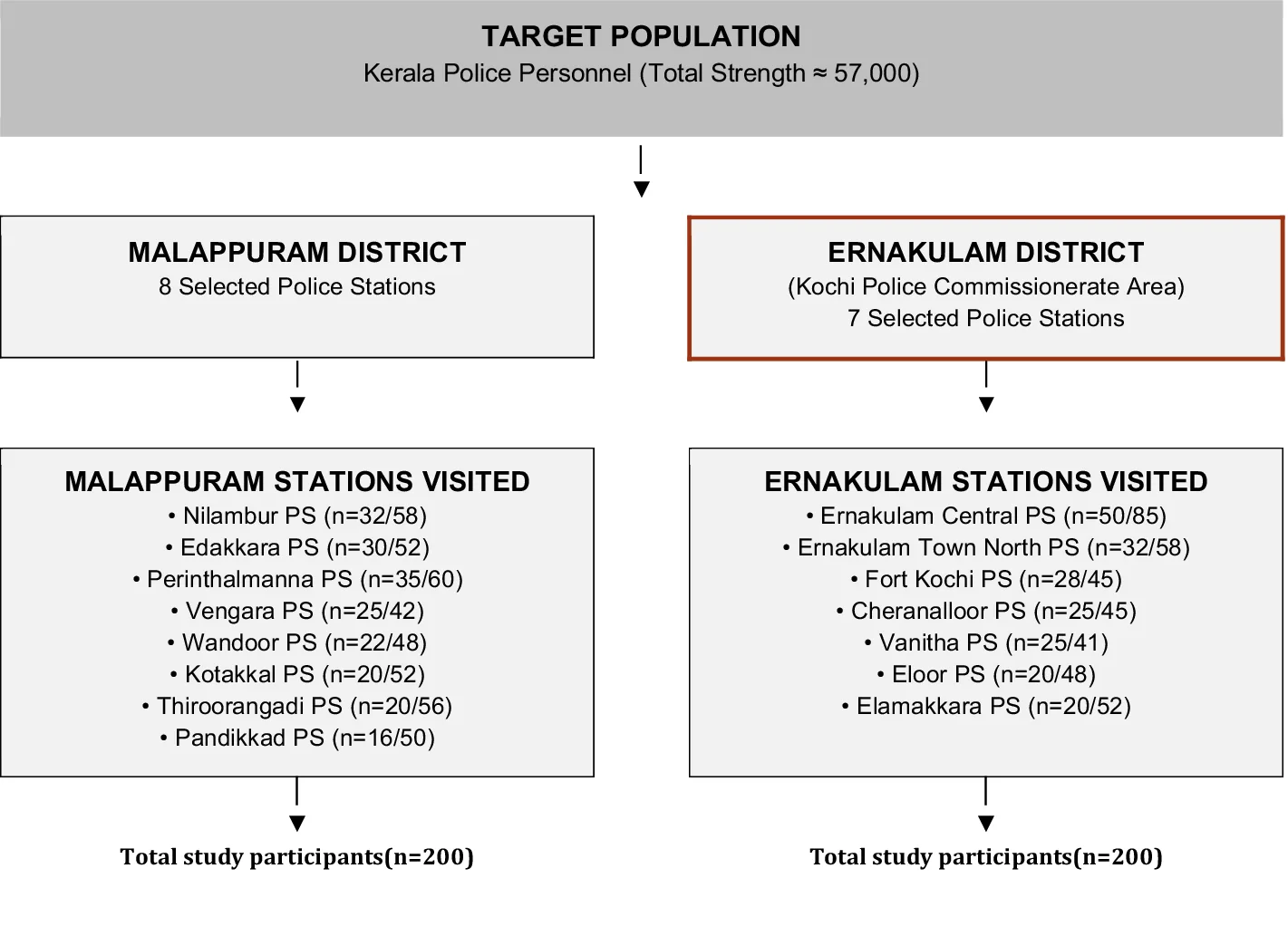
Sampling process.

### Data collection

Data were collected using a pre-tested, self-administered structured questionnaire. After obtaining written informed consent, eligible police personnel were provided with the questionnaire and requested to complete it independently in a private setting within the selected police stations. Trained investigators remained available to clarify any queries without influencing participants’ responses. The completed questionnaires were collected after completion and reviewed for completeness and consistency before data entry and analysis.

### Study tool

Suicidal ideation and suicidal behavior during the past 12 months were assessed using the Columbia-Suicide Severity Rating Scale (C-SSRS), Recent Clinical Version [Version 1/14/09], with the reference period adapted to the past 12 months for the present study. The questionnaire was self-administered by participants in a private setting. Suicidal ideation was assessed across five levels of increasing severity: (1) wish to be dead, (2) non-specific active suicidal thoughts, (3) active suicidal ideation with methods but without intent to act, (4) active suicidal ideation with some intent to act but without a specific plan, and (5) active suicidal ideation with a specific plan and intent. Participants reporting any of these categories during the past 12 months were classified as having suicidal ideation. Suicidal behavior was assessed using the C-SSRS categories of actual suicide attempt, interrupted attempt, aborted or self-interrupted attempt, and preparatory acts or behavior. Participants reporting at least one of these behaviors during the past 12 months were classified as having any suicidal behavior. These categories were not mutually exclusive, and actual suicide attempts were also reported separately from the composite suicidal-behavior outcome.

The C-SSRS was translated from English into Malayalam and back-translated into English to assess consistency with the original version. The translated version was reviewed by experts for clarity, appropriateness, and consistency with the English version, with discrepancies resolved before finalisation. Both English and Malayalam versions were made available, and participants could use the version with which they were comfortable. No separate psychometric validation or reliability assessment of the Malayalam version was undertaken.

### Independent variables

The independent variables included sociodemographic, occupational, and health-related characteristics. Sociodemographic variables comprised age, sex, marital status, educational qualification, type of family, place of residence, religion, district, alcohol consumption and tobacco use, and family history of mental illness. Occupational variables included designation, years of service, average working hours per day, frequency of night duty, type of posting, exposure to traumatic events during police service, availability of psychological support at the workplace, and experience of workplace violence or harassment. Health-related variables included self-reported chronic medical illness and previous psychiatric illness.

Exposure to traumatic or critical incidents was assessed using a binary (yes/no) question, with examples including exposure to violence, death, and disasters. No fixed recall period was specified, and the type, frequency, and timing of traumatic exposures were not assessed separately. Workplace violence or harassment was defined as self-reported exposure to physical, verbal, or psychological violence or harassment at the workplace. Previous psychiatric illness was assessed by asking participants whether they had ever been diagnosed with a psychiatric illness, such as depression or anxiety (yes/no), and therefore represented a self-reported lifetime history of psychiatric diagnosis. Specific diagnoses were not recorded separately or independently verified using medical records.

### Outcome variables

The primary outcome variables were self-reported suicidal ideation and suicidal behavior during the preceding 12 months. Actual suicide attempts were additionally reported as a separate outcome.

### Statistical analysis

Data were entered into Microsoft Excel and analyzed using Stata version 14 (StataCorp, College Station, TX, USA). All analyses accounted for the multistage cluster sampling design. To account for the multistage cluster sampling design, all analyses incorporated station-specific sampling weights, clustering by police station, and stratification by administrative setting. Malappuram District Police and Kochi City Police Commissionerate were specified as strata, and the 15 selected police stations as primary sampling units (PSUs). Sampling weights were calculated as $N_{i}/n_{i}$ , where $N_{i}$ represented the total personnel strength of the selected police station and $n_{i}$ represented the number of participants recruited from that station. No additional normalisation of the weights was performed. The survey design was specified using the *svyset* command, with police station as the PSU, administrative setting as the stratification variable, and station-specific sampling weights as probability weights. Categorical variables were summarized using unweighted frequencies and survey-weighted percentages.

Age was dichotomized at the sample median of 36 years as ≤36 years and >36 years. Professional experience was categorized as ≤15 years and >15 years based on the distribution of years of service in the study population to provide adequately sized groups for analysis. The 15-year cut-off was empirically selected and did not represent a pre-specified or established occupational threshold. Professional designation was described using individual rank categories but dichotomized for regression analysis as Civil Police Officer (CPO) versus other designations (SCPO, ASI, SI, and Inspector). This categorization was adopted to improve analytical stability because of the small and uneven numbers in some rank categories, particularly Inspectors (*n* = 2), which could result in sparse outcome cells and unstable estimates. The combined other-designation category was used as the reference group.

The survey-weighted prevalence of suicidal ideation and suicidal behavior during the past 12 months was estimated with 95% confidence intervals. Actual suicide attempts were additionally reported separately from the composite suicidal-behavior outcome. Bivariable associations between independent variables and each outcome were examined using survey-weighted generalized linear models with a Poisson distribution, log link, and robust variance estimator to estimate unadjusted prevalence ratios (uPRs) and their 95% confidence intervals.

Candidate variables for multivariable analysis were selected based on previous literature, epidemiological and occupational relevance, and availability within the study questionnaire. Variables with a *p*-value <0.20 in bivariable analysis were considered for multivariable analysis, together with variables considered epidemiologically relevant *a priori* irrespective of their bivariable statistical significance. The *p* < 0.20 criterion was therefore used as an initial screening threshold rather than the sole basis for variable selection. Correlation among candidate variables, sparse outcome counts across categories, and model parsimony were also considered during model specification. The final model for suicidal ideation included age, family type, alcohol or other substance use, designation, working hours, chronic medical illness, previous psychiatric illness, and workplace violence or harassment. Because only 14 participants reported suicidal behavior, the model for this outcome was deliberately restricted to four variables-years of service, designation, previous psychiatric illness, and workplace violence or harassment-to reduce the risk of overfitting and model instability. Adjusted prevalence ratios (aPRs) with 95% confidence intervals were reported.

Multicollinearity among candidate variables was assessed using correlation analysis and variance inflation factors (VIFs). Age and years of service were strongly correlated (Pearson’s r = 0.95) and were therefore not included simultaneously in the multivariable models. The choice between these variables was based on their relevance to the respective outcome, bivariable association, and model parsimony. VIFs for variables retained in the final models were <2, indicating no substantial multicollinearity. No adjustment for multiple testing was performed because the analyses were exploratory; accordingly, effect estimates and their 95% confidence intervals were emphasized rather than statistical significance alone. A *p*-value <0.05 was considered statistically significant.

## Results

All 400 enrolled participants were included in the final analysis, with no missing observations for the variables analysed. Therefore, no imputation or other missing-data procedure was required. Among the 400 participants, 197 (50.52%) were aged above 36 years, the mean (SD) age was 37.94 (8.03) years, 323 (80.93%) were male, and 379 (94.34%) were married. Most had undergraduate education [340 (83.92%)], belonged to nuclear families [357 (89.77%)], lived in rural areas [309 (78.33%)], and reported no alcohol consumption or tobacco use [299 (73.65%)], while 10 (2.38%) reported a family history of mental illness (Table 1). Regarding occupational characteristics, 151 (38.40%) were senior CPOs, 250 (62.75%) had ≤15 years of service, 80 (20.06%) reported working more than 12 h per day, and 387 (96.26%) reported weekly night duty. Most reported exposure to traumatic events [390 (97.70%)], 357 (88.60%) reported no availability of psychological support, and 22 (5.86%) reported a previous psychiatric illness (Table 2).

**Table 1:** Sociodemographic characteristics of the study participants (*N* = 400).

Variable	Category	*n*	Weighted %
Age (years)	≤36	203	49.48
	>36	197	50.52
Sex	Female	77	19.07
	Male	323	80.93
Marital status	Single	21	5.66
	Married	379	94.34
Educational qualification	Plus two/Diploma	30	7.95
	Undergraduate	340	83.92
	Postgraduate	30	8.13
Type of family	Joint	43	10.23
	Nuclear	357	89.77
Place of residence	Rural	309	78.33
	Urban	91	21.67
Religion	Christian	107	27.25
	Hindu	221	55.05
	Muslim	72	17.70
District	Ernakulam	200	54.40
	Malappuram	200	45.60
Alcohol and/or tobacco use	Yes	101	26.35
	No	299	73.65
Family history of mental illness	Yes	10	2.38
	No	390	97.62

Values are presented as unweighted frequencies (n) and survey-weighted percentages (%), accounting for the multistage sampling design, clustering by police station, stratification by district, and sampling weights. Plus two = 12 years of schooling.

**Table 2:** Occupational and work-related characteristics of the study participants (*N* = 400).

Variable	Category	*n*	Weighted %
Designation	Civil police officer (CPO)	110	27.41
	Senior civil police officer	151	38.40
	Assistant sub-inspector	85	21.25
	Sub-inspector/	52	13.00
	Inspector	2	0.50
Years of service	≤15 years	250	62.75
	>15 years	150	37.25
Current posting	Law and order	398	99.42
	Special unit	2	0.58
Average working hours/day	≤12 h	320	79.94
	>12 h	80	20.06
Frequency of night duty	Never/Occasionally	13	3.74
	Weekly	387	96.26
Type of posting	Rural	201	45.86
	Urban	199	54.14
Exposure to traumatic events	Yes	390	97.70
	No	10	2.30
Availability of psychological support	Yes	43	11.40
	No	357	88.60
Chronic medical condition	Yes	31	8.14
	No	369	91.86
Previous psychiatric illness	Yes	22	5.86
	No	378	94.14

Values are presented as unweighted frequencies (n) and survey-weighted percentages (%), accounting for the multistage sampling design, clustering by police station, stratification by district, and sampling weights.

During the previous 12 months, 36 participants reported suicidal ideation, corresponding to a weighted prevalence of 9.39% (95% CI: 6.18–14.01). A wish to be dead was the most frequently reported form of suicidal ideation [*n* = 32 (7.81%)], followed by non-specific active suicidal thoughts [*n* = 17 (4.51%)]. Overall, 14 participants reported suicidal behavior during the past 12 months, with a weighted prevalence of 4.11% (95% CI: 1.68–9.70)., The reported behaviors included actual suicide attempts [*n* = 6; 1.47% (95% CI: 0.66–3.25)], interrupted attempts [*n* = 10; 2.89% (95% CI: 1.28–6.42)], aborted or self-interrupted attempts [*n* = 12; 3.60% (95% CI: 1.15–10.71)], and preparatory acts or behavior [*n* = 6; 1.44% (95% CI: 0.51–3.99)] (Table 3). These categories were not mutually exclusive.

**Table 3:** Suicidal ideation and suicidal behavior during the previous 12 months among the study participants based on the Columbia-Suicide Severity Rating Scale (C-SSRS) (*N* = 400).

Domain	Category	Unweighted n	Survey-weighted prevalence (%)	95% CI
Suicidal ideation (five hierarchical items)	Wish to be dead	32	7.81	5.31–11.35
	Non-specific active suicidal thoughts	17	4.51	2.45–8.16
	Active suicidal ideation with any methods (without intent to act)	14	3.71	2.13–6.39
	Active suicidal ideation with some intent to act (without specific plan)	10	2.50	1.58–3.93
	Active suicidal ideation with specific plan and intent	7	1.67	0.86–3.21
Overall suicidal ideation	Any suicidal ideation	36	9.39	6.18–14.01
Severity of suicidal ideation among participants with suicidal ideation (*n* = 36)^a^	Wish to be dead	20	56.17	–
	Non-specific active suicidal thoughts	3	8.47	–
	Active suicidal ideation with any methods (without intent to act)	4	12.93	–
	Active suicidal ideation with some intent to act (without specific plan)	3	8.87	–
	Active suicidal ideation with specific plan and intent	6	13.56	–
Suicidal behavior (four behavioral outcomes)^b^	Actual suicide attempt	6	1.47	0.66–3.25
	Interrupted suicide attempt	10	2.89	1.28–6.42
	Aborted/self-interrupted suicide attempt	12	3.60	1.15–10.71
	Preparatory acts or behavior	6	1.44	0.51–3.99
Overall suicidal behavior	Any suicidal behavior	14	4.11	1.68–9.70

n represents the unweighted number of participants. Prevalence estimates and 95% confidence intervals are survey-weighted and account for sampling weights, clustering by police station, and stratification by administrative setting.

a Severity categories represent the highest level of suicidal ideation reported among the 36 participants with suicidal ideation. Percentages are survey-weighted within this subgroup; 95% CIs were not available for these descriptive severity percentages.

b Suicidal-behavior categories are not mutually exclusive; a participant could report more than one category. Therefore, the individual category frequencies should not be summed. The overall *n* = 14 represents unique participants reporting at least one suicidal behavior during the past 12 months.

Actual suicide attempts are reported separately from the composite outcome of any suicidal behavior.

In the adjusted analysis, participants aged above 36 years reported a higher prevalence of suicidal ideation than those aged ≤36 years (aPR: 2.90; 95% CI: 1.31–6.38). CPOs reported a higher prevalence than personnel in other designations (aPR: 2.31; 95% CI: 1.59–3.37). Working for more than 12 h per day (aPR: 1.67; 95% CI: 1.05–2.65), previous psychiatric illness (aPR: 1.67; 95% CI: 1.19–2.35), and workplace violence or harassment (aPR: 3.16; 95% CI: 1.11–9.04) were also independently associated with a higher prevalence of suicidal ideation than their counterparts (Table 4).

**Table 4:** Bivariable and multivariable analysis of factors associated with suicidal ideation (*N* = 400).

Variable	Suicidal ideation n (w %)	No suicidal ideation n (w%)	uPR (95% CI)	*p* value	aPR (95% CI)	*p* value
Age (years)						
>36 years	25 (13.4)	172 (86.6)	2.55 (0.89–7.35)	0.078	2.90 (1.31–6.38)	0.012
≤36 years	11 (5.3)	192 (94.7)	Ref	–	Ref	–
Sex						
Female	31 (10.1)	292 (89.9)	1.62 (0.56–4.74)	0.347	–	–
Male	5 (6.3)	72 (93.7)	Ref	–	–	–
Marital status						
Unmarried	2 (12.2)	19 (87.8)	1.32 (0.42–4.15)	0.606	–	–
Married	34 (9.2)	345 (90.8)	Ref	–	–	–
Educational qualification						
Plus 2/Diploma	6 (27.8)	24 (72.2)	3.57 (0.91–14.06)	0.066	–	–
Graduate and above	30 (7.8)	340 (92.2)	Ref	–	–	–
Type of family						
Joint	6 (16.1)	37 (83.9)	1.87 (1.02–3.41)	0.043	1.15 (0.60–2.23)	0.65
Nuclear	30 (8.6)	327 (91.4)	Ref	–	Ref	–
Place of residence						
Urban	30 (10.0)	279 (90.0)	1.41 (0.56–3.56)	0.439	–	–
Rural	6 (7.1)	85 (92.9)	Ref	–	–	–
Alcohol and/or tobacco use						
Yes	15 (17.1)	86 (82.9)	2.58 (0.86–7.74)	0.086	1.33 (0.57–3.09)	0.484
No	21 (6.6)	278 (93.4)	Ref	–	Ref	–
Family history of mental illness						
Yes	2 (18.1)	8 (81.9)	1.97 (0.47–8.32)	0.327	–	–
No	34 (9.2)	356 (90.8)	Ref	–	–	–
Designation						
Civil Police Officer (CPO)	14 (13.7)	96 (86.3)	1.76 (0.89–3.47)	0.097	2.31 (1.59–3.37)	<0.001
Other*	22 (7.8)	268 (92.2)	Ref	–	Ref	–
Years of service						
>15 years	18 (12.4)	132 (87.6)	1.64 (0.74–3.62)	0.197	–	–
≤15 years	18 (7.6)	232 (92.4)	Ref	–	–	–
Working hours/day						
>12 h	12 (15.2)	68 (84.8)	1.91 (1.24–2.95)	0.007	1.67 (1.05–2.65)	0.032
≤12 h	24 (7.9)	296 (92.1)	Ref	–	Ref	–
Frequency of night duty						
Never/Occasionally	2 (15.6)	11 (84.4)	1.70 (0.59–4.92)	0.3	–	–
Weekly	34 (9.2)	353 (90.8)	Ref	–	–	–
Exposure to traumatic events						
No	1 (10.5)	9 (89.5)	1.12 (0.19–6.58)	0.891	–	–
Yes	35 (9.4)	355 (90.6)	Ref	–	–	–
Availability of psychological support						
No	32 (9.4)	325 (90.6)	1.03 (0.58–1.81)	0.924	–	–
Yes	4 (9.2)	39 (90.8)	Ref	–	–	–
Chronic medical illness						
Yes	5 (15.7)	26 (84.3)	1.77 (0.94–3.36)	0.075	0.89 (0.51–1.56)	0.659
No	31 (8.8)	338 (91.2)	Ref	–	Ref	–
Previous psychiatric illness						
Yes	6 (28.2)	16 (71.8)	3.43 (2.14–5.49)	<0.001	1.67 (1.19–2.35)	0.006
No	30 (8.2)	348 (91.8)	Ref	–	Ref	–
Experienced workplace violence/harassment						
Yes	21 (20.4)	98 (79.6)	4.34 (1.06–17.84)	0.043	3.16 (1.11–9.04)	0.034
No	15 (4.7)	266 (95.3)	Ref	–	Ref	–

n represents unweighted frequencies. Percentages (w%) are survey-weighted row percentages obtained from svy: tab …, row., others = Senior civil police officer/Assistant sub-inspector/Sub-inspector/Inspector, uPR, unadjusted prevalence ratio; aPR, adjusted prevalence ratio; CI, confidence interval. Variables with *p* < 0.20 in the bivariable analysis and those considered clinically relevant were entered into the multivariable Poisson regression model. To avoid multicollinearity, only one of the correlated variables (e.g., age or years of service, education or designation) was included in the final model. Blank adjusted columns indicate variables not retained in the final multivariable model. *indicates that “Other” under Designation comprises Senior Civil Police Officers, Assistant Sub-Inspectors, Sub-Inspectors, and Inspectors.

As presented in Table 5, the adjusted prevalence of reported suicidal behavior was higher among CPOs than among personnel in other designations (aPR: 12.32; 95% CI: 6.12–24.80) and among participants with more than 15 years of service compared with those with ≤15 years of service (aPR: 13.82; 95% CI: 5.00–38.19). A higher prevalence of suicidal behavior was also observed among participants with a previous psychiatric illness (aPR: 5.94; 95% CI: 2.58–13.66) and those reporting workplace violence or harassment (aPR: 13.81; 95% CI: 2.71–70.34) compared with their respective counterparts.

**Table 5:** Bivariable and multivariable analysis of factors associated with suicidal behavior (*N* = 400).

Variable	Suicidal behavior n (w %)	No suicidal behavior n (w %)	uPR (95% CI)	*p* value	aPR (95% CI)	*p* value
Age (years)						
>36 years	13 (7.5)	184 (92.5)	12.60 (0.93–170.24)	0.056		
≤36 years	1 (0.6)	202 (99.4)	Ref	–		
Sex						
Female	13 (4.7)	310 (95.3)	3.03 (0.21–44.15)	0.387	–	–
Male	1 (1.6)	76 (98.4)	Ref	–	–	–
Marital status						
Unmarried	2 (12.2)	19 (87.8)	3.37 (1.15–9.86)	0.03		
Married	12 (3.6)	367 (96.4)	Ref	–		
Educational qualification						
Plus 2/Diploma	5 (22.9)	25 (77.1)	9.18 (2.39–35.26)	0.003	–	–
Graduate and above	9 (2.5)	361 (97.5)	Ref	–	–	–
Type of family						
Joint	3 (6.0)	40 (94.0)	1.55 (0.22–10.89)	0.633	–	–
Nuclear	11 (3.9)	346 (96.1)	Ref	–	–	–
Place of residence						
Urban	11 (4.5)	298 (95.5)	1.74 (0.14–20.82)	0.64	–	–
Rural	3 (2.6)	88 (97.4)	Ref	–	–	–
Alcohol and/or tobacco use						
Yes	10 (12.4)	91 (87.6)	11.02 (1.75–69.35)	0.014		
No	4 (1.1)	295 (98.9)	Ref	–		
Family history of mental illness						
Yes	1 (7.9)	9 (92.1)	1.97 (0.20–19.16)	0.53	–	–
No	13 (4.0)	377 (96.0)	Ref	–	–	–
Designation						
Civil Police Officer (CPO)	6 (7.2)	104 (92.8)	2.42 (0.88–6.61)	0.08	12.32 (6.12–24.80)	<0.001
Other	8 (3.0)	282 (97.0)	Ref	–	Ref	–
Years of service						
>15 years	9 (6.8)	141 (93.2)	2.73 (0.94–7.92)	0.062	13.82 (5.00–38.19)	<0.001
≤15 years	5 (2.5)	245 (97.5)	Ref	–	Ref	
Working hours/day						
>12 h	6 (7.2)	74 (92.8)	2.15 (0.44–10.58)	0.316	–	–
≤12 h	8 (3.3)	312 (96.7)	Ref	–	–	–
Frequency of night duty						
Never/Occasionally	2 (15.6)	11 (84.4)	4.24 (1.47–12.22)	0.011	–	–
Weekly	12 (3.7)	375 (96.3)	Ref	–	–	–
Availability of psychological support						
No	13 (4.3)	344 (95.7)	1.65 (0.23–12.04)	0.593	–	–
Yes	1 (2.6)	42 (97.4)	Ref	–	–	–
Chronic medical illness						
Yes	2 (5.3)	29 (94.7)	1.32 (0.20–8.50)	0.753	–	–
No	12 (4.0)	357 (96.0)	Ref	–	–	–
Previous psychiatric illness						
Yes	5 (27.5)	17 (72.5)	10.37 (5.78–18.60)	<0.001	5.94 (2.58–13.66)	<0.001
No	9 (2.7)	369 (97.3)	Ref	–	Ref	–
Experienced workplace violence/harassment						
Yes	12 (12.4)	107 (87.6)	21.54 (2.61–177.94)	0.008	13.81 (2.71–70.34)	0.004
No	2 (0.6)	279 (99.4)	Ref	–	Ref	–

n represents unweighted frequencies. Percentages (w%) are survey-weighted row percentages obtained from svy: tab …, row., others = Senior civil police officer/ Assistant sub-inspector/ Sub-inspector/ Inspector, uPR, unadjusted prevalence ratio; aPR, adjusted prevalence ratio; CI, confidence interval. Suicidal behavior included actual suicide attempt, interrupted attempt, aborted or self-interrupted attempt, or preparatory acts or behavior during the past 12 months. Variables with *p* < 0.20 in the bivariable analysis and those considered clinically relevant were entered into the multivariable Poisson regression model. To avoid multicollinearity, only one of the correlated variables (e.g., age or years of service) was included in the final model. Blank cells indicate variables not retained in the final multivariable model.

## Discussion

In this study, approximately one in ten police personnel reported suicidal ideation during the previous 12 months, while one in twenty-five reported a suicide attempt, highlighting that suicidal behavior remains an important occupational mental health concern in this workforce. The findings are particularly relevant in the context of Kerala, which continues to report one of the highest suicide burdens in India. According to the latest National Crime Records Bureau (NCRB), Kerala recorded 10,972 suicides in 2023, corresponding to a suicide rate of 30.6 per 100,000 population, substantially higher than the national average of 12.3 per 100,000 (20). Furthermore, data presented in the Kerala Legislative Assembly showed that 39,962 suicides occurred in the state between January 2021 and March 2025, indicating a persistently high burden of suicide despite improvements in healthcare access and mental health services (21). Police personnel constitute an especially vulnerable occupational group within this context because of their routine exposure to long working hours, operational stress, traumatic incidents, workplace violence, and demanding public interactions. Recent evidence suggests that suicidal behavior among police personnel results from a complex interaction of occupational stressors, organizational factors, and individual vulnerabilities rather than a single precipitating factor (22–24). These findings emphasize the need for occupation-specific suicide prevention strategies within the Kerala Police.

The prevalence of suicidal ideation observed in the present study was lower than that reported in a community-based study among adults in rural and urban Puducherry (19). However, direct comparison of these estimates should be made cautiously because the studies differed in their populations and potentially in the instruments, outcome definitions, recall periods, and sampling approaches used. The Puducherry study represented the general population, whereas the present study included police personnel, who experience a distinct occupational environment. Police work may involve repeated exposure to potentially distressing events, including violence, road traffic fatalities, suicides, disasters, and criminal investigations, together with organizational stressors such as prolonged working hours and demanding duty conditions. Previous studies among police personnel have reported associations of suicidality with traumatic exposures, organizational stress, burnout, limited psychological support, and other occupational factors (22–24). Krishnan et al. (23) highlighted the potential importance of cumulative traumatic exposure, organizational stress, burnout, inadequate recovery, and limited psychological support, while Chae and Boyle (24) described the possible roles of organizational culture, chronic occupational stress, and barriers to recognizing psychological distress in police suicide. An Indian study by Patel also reported an association between occupational stress and suicide risk among police personnel (22). These findings provide possible explanations for the associations observed in the present study; however, the underlying mechanisms were not directly examined, and the cross-sectional design does not permit causal inference.

Older age was independently associated with a higher reported prevalence of suicidal ideation, while personnel with more than 15 years of service reported a substantially higher prevalence of suicidal behavior. Although age and duration of service are closely correlated, they may reflect different aspects of cumulative occupational exposure. Older personnel often assume greater professional responsibilities and may experience prolonged exposure to stressful situations, repeated traumatic events, shift work, and organizational pressures accumulated over several years. Such chronic exposure may progressively reduce psychological resilience and increase vulnerability to suicidal behavior. Similar observations were reported by Violanti et al. (25), who demonstrated that cumulative occupational stress and hopelessness were strongly associated among police personnel. Furthermore, Cranney and Wilkinson (26) showed that suicide risk increases with advancing age in the presence of persistent psychosocial stressors and inadequate coping resources, particularly among working populations. These findings highlight the importance of regular psychological screening and targeted mental health interventions throughout the occupational lifespan of police personnel.

CPOs reported a higher prevalence of both suicidal ideation and suicidal behavior than personnel in senior designations. CPOs generally perform frontline duties involving continuous public interaction, emergency response, law-and-order maintenance, and conflict management, thereby exposing them to greater operational stress than personnel in supervisory or administrative roles. These responsibilities often involve unpredictable situations requiring rapid decision-making under pressure while providing limited opportunities for recovery. Previous evidence has consistently shown that frontline policing is associated with greater occupational stress, burnout, and psychological morbidity than higher-ranking positions (23, 24). In addition, Chen et al. observed that reduced social and organizational support in demanding occupational settings may contribute to poorer mental health outcomes and increased suicide risk (27). The present findings therefore suggest that frontline police personnel should constitute a priority group for workplace mental health promotion and suicide prevention initiatives.

Working for more than 12 h per day was independently associated with a higher reported prevalence of suicidal ideation. Long working hours may contribute to chronic fatigue, sleep deprivation, burnout, impaired work-life balance, and reduced coping capacity, all of which adversely affect psychological well-being. Sleep disturbance has consistently been recognized as an important pathway linking occupational stress to suicidal thoughts among public safety personnel. Plant et al. (28) demonstrated that sleep disturbances significantly predicted suicidal thoughts and behaviors among police and other public safety personnel. Likewise, Di Nota et al. (29) reported that chronic occupational stress, together with mental health disorders, substantially increased the risk of suicidal ideation and suicide attempts among Canadian police personnel. These findings support organizational interventions aimed at reducing excessive workloads, ensuring adequate rest periods, and promoting healthy work schedules as important strategies for improving mental health within police organizations.

Previous psychiatric illness and workplace violence or harassment emerged as consistent factors of both suicidal ideation and suicidal behavior. Police personnel with a previous psychiatric illness reported significantly higher prevalence of both outcomes, emphasizing the importance of early identification, timely treatment, and continued follow-up of mental health conditions within the police force. Similar associations between psychiatric disorders and suicidal behavior have been consistently reported among police personnel (30, 31). Participants who experienced workplace violence or harassment also reported substantially higher prevalence of suicidal ideation and suicidal behavior. Workplace violence may contribute to persistent psychological distress, emotional exhaustion, reduced job satisfaction, and feelings of isolation, thereby increasing vulnerability to suicidal behavior. Recent evidence suggests that psychologically unsafe work environments, bullying, and workplace harassment adversely affect mental health and may increase suicide risk among high-stress occupations (32, 33). Together, these findings indicate that suicide prevention programs for police personnel should combine individual-level mental health interventions with organizational measures to prevent workplace violence, foster psychologically safe work environments, encourage confidential reporting of mental health concerns, and improve access to professional psychological support.

The findings of the present study have important public health implications for Kerala. The observed associations should not be interpreted as evidence of causal relationships. Although several occupational and mental health factors were associated with a higher prevalence of suicidal ideation or suicidal behavior, the cross-sectional design does not establish their temporal sequence. Potential explanations discussed above, including cumulative occupational stress, psychological consequences of workplace violence or harassment, and barriers to seeking mental health support, represent plausible mechanisms based on previous literature and were not directly examined in the present study. The state has strengthened community-based mental health services through initiatives such as the District Mental Health Program (DMHP), the Aswasam program for early detection and management of depression, Tele-*MANAS*, and the Kerala State Mental Health Authority, which collectively aim to improve access to mental health care and suicide prevention services (34–36). Despite these initiatives, police personnel represent a high-risk occupational group with unique workplace stressors that require targeted organizational interventions. Based on the findings of this study, routine confidential mental health screening, timely identification and treatment of personnel with previous psychiatric illness, measures to reduce excessive working hours, prevention of workplace violence and harassment, and regular psychological assessment of personnel with longer service duration should be prioritized. In addition, strengthening peer-support programs, promoting mental health literacy among supervisors, ensuring confidential access to psychological counseling, and integrating occupation-specific mental health services into existing Kerala Police welfare programs may help reduce suicidal behavior and improve the psychological well-being of police personnel.

A major strength of this study is the use of a validated instrument to assess suicidal ideation and suicidal behavior, together with survey-weighted analyses accounting for the multistage cluster sampling design. However, the findings should be interpreted in light of several limitations. The cross-sectional design precludes establishing temporality or causality between the factors examined and suicidal ideation or suicidal behavior, and reverse causation cannot be excluded. Therefore, the observed associations should not be interpreted as evidence that the identified factors preceded or caused suicidal outcomes. Information on suicidal ideation, suicidal behavior, previous psychiatric illness, workplace violence or harassment, substance use, and other study variables was self-reported and may therefore be subject to recall and social-desirability bias. Despite measures to ensure privacy and confidentiality, the sensitive nature of these questions and concerns regarding mental health stigma or occupational consequences may have resulted in under-reporting and consequent underestimation of some outcomes and exposures.

Individual-level recruitment was based on the availability of eligible personnel during data-collection visits rather than random selection. The numbers of eligible personnel who were absent, declined participation, or otherwise did not participate, and their reasons for non-participation, were not systematically documented separately. An exact response rate could therefore not be calculated, and the characteristics of participants could not be compared with those of non-participants. Consequently, non-response and availability-related selection bias cannot be excluded. Although station-specific sampling weights accounted for unequal sampling fractions across the selected police stations, they cannot fully address potential selection arising from availability-based recruitment at the individual level.

The study included only 15 police stations from two administrative settings-Malappuram District Police and Kochi City Police Commissionerate-which were not randomly selected from all police administrative units in Kerala. The limited number of PSUs also resulted in limited survey design degrees of freedom, potentially reducing the precision of variance estimates. Personnel from other districts, Commissionerate’s, specialized units, and those unavailable during data collection may differ in their occupational exposures, working conditions, and access to support services. The findings should therefore be generalized to the broader Kerala Police population with caution. Suicidal behavior was reported by only 14 participants, resulting in sparse outcome data and wide confidence intervals for several estimates. Although the multivariable model was deliberately restricted to four variables to reduce overfitting, sparse-data bias and model instability cannot be excluded. The large adjusted prevalence ratios observed for some factors may therefore overestimate the magnitude of the underlying associations and should be considered exploratory rather than definitive independent effects.

Nearly all participants reported exposure to traumatic or critical incidents, resulting in limited variability and reducing the ability to examine its association with suicidal outcomes. Moreover, the type, frequency, timing, and recall period of traumatic exposures were not assessed in detail. Although recent significant life stressors and perceived social support were assessed, these constructs were measured using brief questionnaire items rather than comprehensive validated scales. Dedicated validated instruments were also not used to assess potentially important psychological factors such as depression, anxiety, post-traumatic stress disorder, burnout, perceived stress, and hopelessness. Other factors, including coping mechanisms, personality characteristics, and financial stress, were not comprehensively assessed. Residual confounding by these unmeasured or incompletely measured factors therefore cannot be excluded. Future longitudinal studies involving larger and more geographically and organizationally diverse samples are needed to clarify the temporal relationships between occupational and psychological factors and suicidal outcomes among police personnel. Such studies should incorporate comprehensive validated measures of psychological and occupational factors. Person-centered approaches, such as latent class analysis, may also help identify distinct risk profiles among police personnel.

## Conclusion

Nearly one in ten police personnel reported suicidal ideation and one in twenty-five reported suicidal behavior during the past 12 months. Older age, longer duration of service, CPO designation, extended working hours, a history of psychiatric illness, and exposure to workplace violence or harassment were each independently associated with a higher reported prevalence of suicidal behaviors. These findings highlight the need for comprehensive workplace mental health programs that promote early identification of psychological problems, reduce occupational stressors, prevent workplace violence, and improve access to confidential mental health services for police personnel.

## Data Availability

The raw data supporting the conclusions of this article will be made available by the authors, without undue reservation.
